# Antidepressants as Autophagy Modulators for Cancer Therapy

**DOI:** 10.3390/molecules28227594

**Published:** 2023-11-14

**Authors:** Leping He, Yuanfeng Fu, Yuxi Tian, Xiaofeng Wang, Xuejun Zhou, Ren-Bo Ding, Xingzhu Qi, Jiaolin Bao

**Affiliations:** 1 Key Laboratory of Tropical Biological Resources of Ministry of Education, School of Pharmaceutical Sciences, Collaborative Innovation Center of One Health, Hainan University, Haikou 570228, China; hlprin98@126.com (L.H.); yuanfeng0414yf@163.com (Y.F.); a18280572994@163.com (Y.T.); dingrenbo@hainanu.edu.cn (R.-B.D.); qxz_hainu_edu@163.com (X.Q.); 2 Department of Otolaryngology-Head and Neck Surgery, The First Affiliated Hospital of Hainan Medical University, Haikou 570102, China; xiaofengwang@hainmc.edu.cn (X.W.); xuejunzhouent@hainmc.edu.cn (X.Z.); 3 State Key Laboratory of Quality Research in Chinese Medicine, Institute of Chinese Medical Sciences, University of Macau, Macao 999078, China

**Keywords:** antidepressants, cancer therapy, drug repurposing, autophagy, molecular mechanism

## Abstract

Cancer is a major global public health problem with high morbidity. Depression is known to be a high-frequency complication of cancer diseases that decreases patients’ life quality and increases the mortality rate. Therefore, antidepressants are often used as a complementary treatment during cancer therapy. During recent decades, various studies have shown that the combination of antidepressants and anticancer drugs increases treatment efficiency. In recent years, further emerging evidence has suggested that the modulation of autophagy serves as one of the primary anticancer mechanisms for antidepressants to suppress tumor growth. In this review, we introduce the anticancer potential of antidepressants, including tricyclic antidepressants (TCAs), tetracyclic antidepressants (TeCAs), selective serotonin reuptake inhibitors (SSRIs), and serotonin-norepinephrine reuptake inhibitors (SNRIs). In particular, we focus on their autophagy-modulating mechanisms for regulating autophagosome formation and lysosomal degradation. We also discuss the prospect of repurposing antidepressants as anticancer agents. It is promising to repurpose antidepressants for cancer therapy in the future.

## 1. Antidepressants in Cancer Therapy

Cancer is a major global public health problem and the second leading cause of death worldwide [1,2]. According to the most recent estimate from the International Agency for Research on Cancer (IARC), there were 10.0 million cancer deaths and 19.3 million new cases worldwide in 2020. By 2040, it is estimated that the number of new cancer patients will increase to 30.2 million, which almost doubles the current level [1,2].

Depression is known to be an important complication of cancer diseases whose prevalence in cancer patients is much higher in these patients than in the healthy population. The incidence of major depressive disorder in the overall population is 3.3%, while in the cancer population, it increases approximately fourfold to 12.5% [3]. Cancer-associated depression decreases patients’ quality of life [4] and compliance with anticancer treatment [5] and increases physical distress [6] and the risk of suicide [7]. A meta-analysis showed that depression could increase the mortality rate of cancer patients by 39%; even if patients experienced only some depressive symptoms, their risk of death may increase by 26% [8]. Therefore, antidepressants are often used as a complementary treatment during cancer therapy.

Over the past 30 years, a series of studies have demonstrated, in vitro and in vivo, the anticancer properties of antidepressants, whose underlying mechanisms include triggering apoptosis, restricting cellular energy metabolism, exhibiting antioxidant activity, inhibiting angiogenesis, regulating the immune system, and so on [9,10,11]. The anticancer antidepressants encompass tricyclic antidepressants (TCAs), tetracyclic antidepressants (TeCAs), selective serotonin reuptake inhibitors (SSRIs), and serotonin-norepinephrine reuptake inhibitors (SNRIs) [9,10,11]. Further studies also have shown that the combination of antidepressants and anticancer drugs affects the effectiveness of cancer treatment by enhancing the antitumor cytotoxic effect and overcoming therapeutic resistance [9,10,11]. Based on the existing findings, repurposing antidepressants represents a good strategy for anticancer drug development. In recent years, emerging evidence has suggested that the modulation of autophagy serves as one of the primary anticancer mechanisms for antidepressants to suppress tumor growth, which attracts much attention from pharmacologists.

With the large demand for new effective anticancer therapeutics and the increasing cost of novel drug development, repurposing antidepressants as anticancer drugs represents a promising strategy. In this review, we introduce the anticancer potential of antidepressants, specifically focusing on their autophagy-modulating mechanisms, and discuss the prospects of repurposing antidepressants as anticancer drugs.

## 2. Autophagy in Cancer

Autophagy is a highly conserved cellular process that maintains cellular homeostasis by degrading and recycling damaged, long-lived, or misfolded proteins, as well as damaged or abnormal organelles [12,13,14]. Under basal conditions, autophagy functions to eliminate damaged organelles and protein aggregates [9]. Autophagy also serves as the energy source for cells under stress conditions such as starvation, hypoxia, and infection [13]. In mammalian cells, several forms of autophagy have been described, including macroautophagy, mitophagy, and chaperone-mediated autophagy [15]. To recognize the importance of autophagy, the discovery of autophagy mechanism was awarded with the Nobel Prize for Physiology or Medicine to Yoshinori Ohsumi in 2016.

Autophagy is a multi-step process of sequential events including initiation, the nucleation of the autophagosome, the maturation and elongation of the autophagosome membrane, and the fusion of the autophagosome with the lysosome, concluding with the degradation and recycling of intravesicular products [16]. The execution of autophagy depends on the control of autophagy-related genes (ATGs). By modulating nutrient, energy, and stress-sensing signaling, ATGs regulate the autophagic process in cells [16]. Once autophagy is activated, a series of ATG protein complexes coordinate to form double-membrane vesicles called autophagosomes that capture “cargo” in the cytoplasm. These “cargoes” usually are damaged or excess proteins, organelles, lipids, and glycogen, which are tagged with ubiquitin and recognized by the autophagic receptor. Cargo receptors bind cargo and the autophagosomal membrane component LC3-II to facilitate cargo sequestration. The fusion between the autophagosome and lysosome provides hydrolase that degrades cargo. The produced amino acids, lipids, nucleosides, and carbohydrates are then released into the cytoplasm for recirculation [17].

In regulating cancer development, autophagy plays different roles depending on the type, stage, or genetic background of a tumor [18,19,20,21,22]. On the one hand, triggering autophagy can restrict the accumulation of oncogenic mutations, limit chromosomal instability, alleviate oxidative stress, and decrease local inflammation. This prevents tumor initiation, proliferation, invasion, and metastasis [16]. In this context, autophagy acts as a tumor-suppressive mechanism, especially in the early stages of tumorigenesis [23,24]. On the other hand, once the tumor progresses to an advanced stage, autophagy activation can work as a protective and defensive mechanism to maintain cellular mitochondrial function and supports the metabolic demands of proliferating tumor cells and enhancing tumor resistance to stress, thereby facilitating tumor progression and inducing resistance to therapeutic drugs [13]. Furthermore, autophagy can also contribute to cancer aggressiveness by promoting invasion and metastasis [25]. Under different circumstances, both appropriate pharmacological induction and inhibition can effectively suppress tumor growth and metastasis.

## 3. Antidepressants Modulate Autophagy for Tumor Therapy

### 3.1. Tricyclic Antidepressants (TCAs)/Tetracyclic Antidepressants (TeCAs)

**Figure 1:**
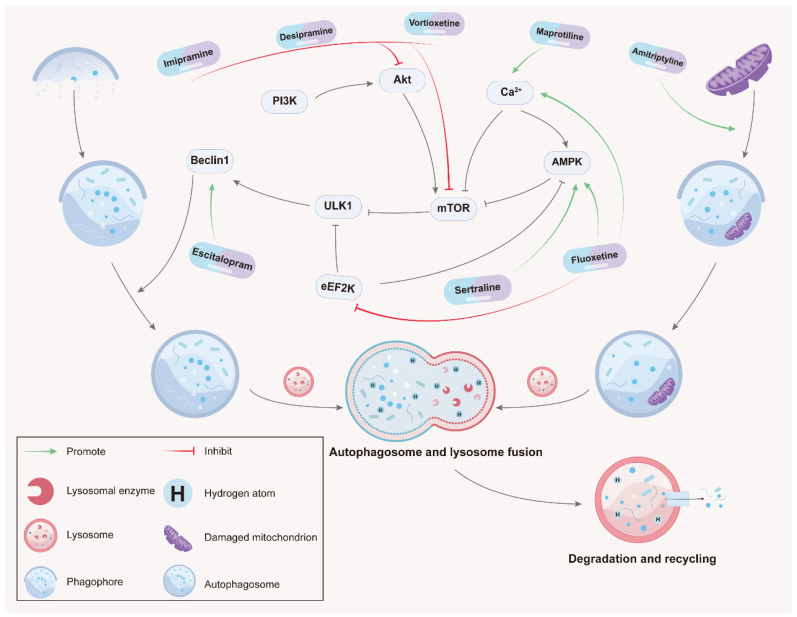
Antidepressants play an anticancer role through inducing autophagy. In cancer cells, imipramine, desipramine, and vortioxetine induce autophagy through inhibiting Akt/mTOR. Maprotiline and fluoxetine enhance autophagy through regulating Ca^2+^ flux followed by AMPK phosphorylation and mTOR inhibition. Fluoxetine and sertraline induce autophagic flux by promoting AMPK-mediated autophagy and inhibiting eEF2K. Escitalopram stimulates Beclin 1 to launch autophagy induction. In addition, amitriptyline induces mitochondrial dysfunction and oxidative stress induces mitophagy.

**Figure 2:**
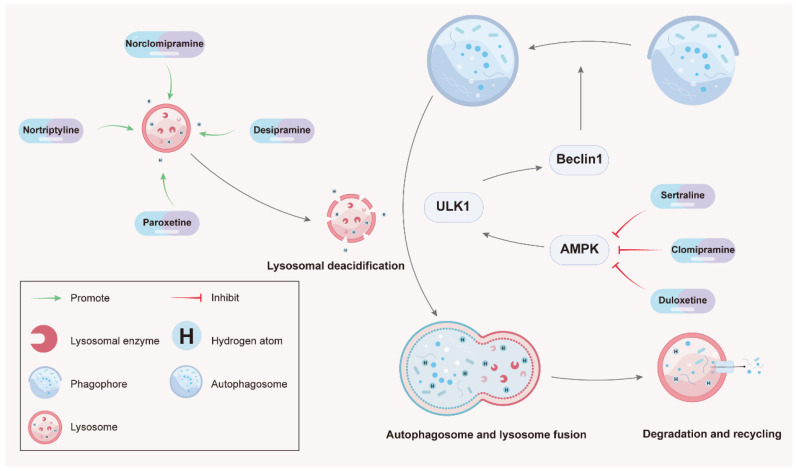
Antidepressants exert anticancer activity through blocking autophagic flux. The autophagy inhibitions by sertraline, clomipramine, and duloxetine take place through activating AMPK. Meanwhile, nortriptyline, norclomipramine, desipramine, and paroxetine inhibit autophagy mediating via lysosomal deacidification and destruction.

Tricyclic and tetracyclic antidepressants, also called cyclic antidepressants, are composed of a central three-ring or four-ring molecular structure with a unique side chain. Recently, in addition to their traditional use in the treatment of psychiatric disorders, TCAs and TeCAs have been reported to exhibit great potential in cancer treatment through modulating autophagy. Studies have shown that imipramine, desipramine, and maprotiline could induce autophagy to suppress cancer (Figure 1 and Table 1) while other studies have demonstrated that amoxapine, clomipramine, desipramine, and nortriptyline are able to inhibit autophagy to block tumor growth (Figure 2 and Table 2).

#### 3.1.1. Imipramine

In glioblastoma, imipramine could induce autophagy with the conversion of LC3-I to LC3-II and the redistribution of LC3 to the autophagosome [26]. It is well known that PI3K/Akt/mTOR signaling is one of the pivotal upstream pathways of autophagy and mTOR plays a crucial role in negatively regulating autophagy by phosphorylating Atg13, which is involved in the activation of the class III PI3K Vps34 [17,51,52]. Imipramine was found to inhibit PI3K/Akt/mTOR pathways and downregulate mTOR phosphorylation to trigger autophagic cell death, but not apoptosis, in human U-87MG glioblastoma cells. Knockdown of Beclin-1 to disrupt the Beclin-1-Vps34 complex could abrogate the imipramine-induced autophagy and cell death [26]. Similar autophagy induction by imipramine was also confirmed by Justice et al. in primary human pulmonary artery endothelial cells, in which the pharmacological autophagy-inducing mechanism of imipramine occurred through the inhibition of acid sphingomyelinase and lysosomal nutrient-sensing-complex-mediated mTOR phosphorylation [53]. In another study, Shchors et al. found that imipramine activated adenylate cyclase and induced cAMP-mediated autophagy, which resulted in autophagy-associated glioblastoma cell death and the prolonged survival of glioma-bearing mice [27]. Combining imipramine with ticlopidine, a P2Y12 inhibitor, could coordinately upregulate intracellular cAMP levels to elicit hyper-activated autophagy and consequent cell death in glioma [27]. Synergistic autophagy induction was also shown by the combination of imipramine and anti-VEGF therapy. Chryplewicz et al. found that the combination of imipramine and B20S, an anti-VEGF antibody, synergistically enhanced the autophagy-dependent recruitment of CD8 and CD4 T cells to promote immunity in glioma-bearing mice and effectively blocked tumor progression [28]. This survival benefit generated from imipramine and anti-VEGF co-treatment could be abrogated by silencing the expression of ATG3, a key regulator of autophagy that is associated with reduced cytotoxic T cell infiltration, revealing the importance of induced autophagic flux in immune cell recruitment [28].

#### 3.1.2. Desipramine

Desipramine is the N-demethylated metabolite of imipramine and is reported to exhibit a similar autophagy induction phenomenon during cancer therapy [29]. Ma et al. demonstrated that desipramine could trigger autophagic glioma death characterized by autophagosome formation, the increased autophagic protein level of Beclin-1, and the cellular distribution of autophagic marker LC3-II. This desipramine-induced autophagy induction was mediated by inhibiting the PI3K-AKT-mTOR pathway and activating the PERK-eIF2α-ER stress pathway while the knockdown of PERK could significantly abolish the autophagy initiated by desipramine, indicating the necessity of PERK-mediated ER stress involvement [29]. As a major signal-transducing organelle, the endoplasmic reticulum (ER) senses and responds to changes in homeostasis [54,55,56]. When the ER is stressed, unfolded protein response (UPR) pathways are activated through the induction of protein kinase RNA-like endoplasmic reticulum kinase (PERK). The α subunit of eukaryotic initiation factor 2 (eIF2α) phosphorylation, which is activated by PERK, inhibits protein synthesis. PERK-eIF2α promotes the induction of ER stress-induced apoptosis [54,55,56]. Interestingly, an inconsistent report was demonstrated by Kuzu et al., in which desipramine acted as an acid sphingomyelinase inhibitor and could block autophagic flux in UACC903 metastatic melanoma cells by inhibiting intracellular cholesterol transport but still effectively restricted melanoma tumor growth [42]. This result was also contradictory to the autophagy induction phenotype of acid sphingomyelinase inhibition mediated by imipramine treatment [53]. However, these studies were conducted in different models, whose heterogenous backgrounds might have contributed to the variations that occurred.

#### 3.1.3. Maprotiline

It has been shown that maprotiline could inhibit glioma cell proliferation since the 1990s [57,58], but its underlying mechanism against cancer was not fully understood for a long time. In 2004, Hsu et al. found that maprotiline inhibited the proliferation of PC3 human prostate cancer cells by regulating intracellular Ca^2+^ influx and release [59]. The anticancer mechanism of maprotiline was further explored by Cloonan and Williams in the chemoresistant DG-75 Burkitt lymphoma cells, which are apoptosis-defective tumor cells lacking the expression of the proapoptotic proteins Bax and Bak [30]. Maprotiline treatment resulted in autophagic programmed cell death in DG-75 cells, which were associated with increases in autophagic vesicles, autophagosome formation, and Beclin-1 expression levels. Such maprotiline-induced pro-autophagic cell death could be rescued by autophagy inhibitors, demonstrating that autophagy induction is primarily responsible for DG-75 cell death treated with maprotiline [30].

#### 3.1.4. Amitriptyline

There are controversial effects of amitriptyline on modulating autophagy among different types of cancer [31,43]. In HepG2 hepatocellular carcinoma cells, amitriptyline induced an early autophagic activation associated with mitochondria dysfunction and oxidative stress and triggered Parkin-mediated mitophagy. Following persistent and extensive mitochondria autophagic stress, amitriptyline subsequently led to mitochondrial toxicity and apoptotic cell death [31]. Controversially, in A549 lung cancer cells, amitriptyline upregulated the expression of both LC3-II and p62, indicating that there was the complete formation of an autophagosome, but the fusion of the autophagosome with lysosome was blocked in the late stage of autophagy flux. TRAIL (tumor necrosis factor-related apoptosis-inducing ligand) is a cytokine that can induce apoptosis in cancer cells while causing minimal toxicity to normal cells. As a consequence, cancer cells that are resistant to TRAIL pose a major challenge for the development of cancer treatments, and to develop drugs that enhance the effectiveness of TRAIL or overcome its resistance to cancer cells is necessary [60]. The amitriptyline-induced autophagy blockage increased DR4 and DR5 expression, subsequently enhancing TRAIL-mediated apoptotic cell death [43].

#### 3.1.5. Nortriptyline

Nortriptyline is an active metabolite of amitriptyline. It has been shown that nortriptyline suppresses autophagic flux, causing the aggregation of the autophagosome and disruption of cancer cell cholesterol homoeostasis, and by inhibiting acid sphingomyelinase, it consequently resulted in the inhibition of major oncogenic signaling cascades on which cancer cells were reliant for survival. In the UACC903 melanoma tumor xenograft model, nortriptyline at a concentration of 5 mg/mL was sufficient to inhibit tumor growth by 50% [42]. Consistently, Chung et al. showed that nortriptyline inhibited autophagic flux by disrupting the lysosome and impeding autolysosome function, leading primarily to non-apoptotic pineoblastoma cell death [44]. Gemcitabine previously was demonstrated to induce autophagy as a protective mechanism for cancer cells, which was abolished by autophagy inhibitors [61,62]. Nortriptyline as an autophagy inhibitor, was reported to further synergize with gemcitabine to suppress pineoblastoma growth [44].

#### 3.1.6. Clomipramine

The use of clomipramine as a clinical drug has lasted for over 40 years and provided well-tolerated toxicity results in subjects of various medical conditions, even in cancer patients [63]. In recent years, clomipramine has been found to exhibit an anticancer property by regulating autophagic fluxes against various types of cancer, including breast, prostate, bladder, cervical, and lung cancer [45,46,64,65]. Among these studies, clomipramine functioned as an autophagy inhibitor. Nguyen et al. reported that clomipramine arrested the fusion of the autophagosome to the lysosome via suppressing AMPK activation and sensitized enzalutamide response in castration-resistant prostate cancer in vitro and in vivo [45]. Furthermore, clomipramine also inhibited autophagy by blocking autophagolysomal fluxes and thus potentiated the therapeutic responses of gemcitabine and mitomycin in a panel of breast, prostate, bladder, and cervical cancer cell lines [65]. Similar observations were demonstrated with an active metabolite of clomipramine, named norclomipramine or desmethylclomipramine, which interfered with autophagic flux by increasing LC3-II but concomitantly blocking the degradation of autophagic cargo [46,64]. Norclomipramine was able to enhance the cytotoxic effect of doxorubicin in Hela cancer cells and cisplatin, gemcitabine, and paclitaxel in lung cancer stem cells in an autophagy-dependent manner [46,64].

### 3.2. Selective Serotonin Reuptake Inhibitors (SSRIs)

The selective serotonin reuptake inhibitors, such as fluoxetine, escitalopram, sertraline, vortioxetine, and paroxetine, are commonly used for the treatment of depression in patients with cancer. It has been documented that SSRIs could induce tumor cell death in various cancer models. In particular, SSRIs have been reported as autophagy modulators to suppress cancer growth in breast cancer, lung cancer, gastric cancer, hepatocellular carcinoma, leukemia, prostate cancer, and many other cancer types (Figure 1 and Figure 2; Table 1 and Table 2).

#### 3.2.1. Fluoxetine

Fluoxetine is the most reported SSRI with an autophagy-modulating property in multiple types of cancer, including Burkitt’s lymphoma [30], breast cancer [32], gastric adenocarcinoma, and lung cancer tumor cells. It was first reported by Cloonan and Williams in 2011, showing that fluoxetine induced Type II autophagic cell death in DG-75 Burkitt’s lymphoma cells [30]. Afterwards, a few further studies demonstrated that fluoxetine induced cytotoxic cell death in triple-negative breast cancer by triggering persistent autophagy via activating the AMPK/mTOR/ULK axis [32,33]. The eukaryotic elongation factor 2 kinase (eEF2K) was reported to play an essential role in the crosstalk between autophagy and apoptosis [66,67], and the inhibition of eEF2K in fluoxetine-treated triple-negative breast cancer was associated with AMPK/ULK-dependent autophagy to promote autophagic and apoptotic cancer cell death simultaneously [33]. Fluoxetine inhibited eEF2K activity by decreasing its phosphorylation at ser78 and ser398, subsequently inducing the AMPK/mTOR/ULK complex pathway and autophagic cell death [33]. Meanwhile, fluoxetine also was found to induce cellular ER stress by promoting the PERK/eIF2α/NF-κB pathway, which was considered to be an important inducement of autophagic cell death [32]. In gastric and lung cancer, fluoxetine significantly caused cancer cell death associated with triggering autophagosome formation with a high accumulation of LC3-II; however, the autophagic degradation process was blocked by fluoxetine as indicated by continuous p62 increase [34,35]. These studies revealed that fluoxetine plays complex roles in autophagic flux modulation among different cancer types; however, its anticancer effect is consistently confirmed.

#### 3.2.2. Escitalopram

Escitalopram is the S-enantiomer of citalopram and is currently used to treat major depressive disorder and anxiety disorder. It has been shown with autophagy-inducing activity in malignant gliomas cells. Obviously, an increased LC3-II/I ratio; the expression of autophagy markers Beclin-1, ATG3, ATG5, and ATG7; and a declined p62 protein level were observed in GBM8401 cells treated with escitalopram [36]. Consistent evidence was further provided with hepatocellular carcinoma models by Chen et al., who demonstrated that escitalopram significantly suppresses the proliferation of HepG2 and Huh-7 cells and tumor growth of Huh-7 xenografts by activating autophagic flux [37]. More importantly, according to the large-cohort epidemiology study investigating the association between liver cancer risk and escitalopram, conducted by Chen et al., patients who used escitalopram had a significantly decreased incidence of liver cancer than those who had never used escitalopram [37].

#### 3.2.3. Sertraline

Sertraline has shown discrepant effects on modulating autophagy in different types of cells. On the one hand, sertraline has been reported to induce autophagic flux among acute myeloid leukemia cells [38], non-small lung cancer cells [40], and prostate cancer stem cells [39]. On the other hand, sertraline has also inhibited autophagy in lung cancer cells [47]. Sertraline can induce a significant increase in autophagic vacuoles with a double-membrane structure, which further facilitates apoptosis in NB4 acute myeloid leukemia cells. A similar observation has also been found in primary acute myeloid leukemia cells [38]. Autophagy blockage partially attenuates sertraline-induced apoptosis and cancer proliferation inhibition in acute myeloid leukemia cells [38]. Consistent results showing sertraline-induced autophagy and apoptosis were demonstrated in prostate cancer stem cells by Chinnapaka et al. [39]. Jiang et al. also confirmed the autophagy-induction and growth-inhibition effects of sertraline; however, they found, differently, that sertraline did not trigger caspase-mediated apoptosis except autophagic cell death in non-small cell lung cancer [40]. Furthermore, they discovered the synergistic tumor-killing effect of sertraline and erlotinib co-treatment in vitro and in vivo by reciprocally regulating the AMPK/mTOR/S6K pathway to reinforce autophagy activation in non-small lung cancer cells. While blocking autophagy, either sertraline alone or its combination with erlotinib was less effective in combating cancer [40]. Interestingly, there have been contradictory findings observed in TRAIL-resistant lung cancer cells. Zinnah et al. reported that sertraline blocked autophagic flux and induced TRAIL-mediated apoptosis via the downregulation of AMPK phosphorylation and upregulation of DR5 expression in lung cancer cells [47].

#### 3.2.4. Vortioxetine

Vortioxetine has been shown to restrain cancer development in multiple aspects including the inhibition of cancer cell proliferation, invasion, and migration. Its pharmacological mechanism has been reported to be associated with simultaneous autophagy and apoptosis induction [41]. In a study, vortioxetine increased the levels of pro-autophagic LC3-II, Beclin-1, proapoptotic Bax, and active Caspase-3/9 and downregulated p62 and Bcl-2 in gastric cancer cells, which was mediated by the suppression of the PI3K-AKT-mTOR pathway [41].

#### 3.2.5. Paroxetine

Paroxetine and its structural derivative N-methylparoxetine were both found to block autophagic flux at the late stage and simultaneously induce mitochondrial fragmentation and ROS overproduction in non-small cell lung cancer cells [48,49]. Specifically, the autophagy inhibition induced by paroxetine and N-methylparoxetine occurred by disrupting lysosomal acidification and altering the maturation lysosomal cathepsins rather than interfering with autophagosome–lysosome fusion. Consequently, the clearance of damaged mitochondria and accumulated ROS by the autophagic process was blocked, which in turn served to activate P38-MAPK and JNK-MAPK cascades and triggered mitochondria-dependent apoptosis, leading to significant growth inhibition in non-small cell lung cancer [48,49].

### 3.3. Serotonin-Norepinephrine Reuptake Inhibitors (SNRsI)

Duloxetine is a serotonin-norepinephrine reuptake inhibitor (SNRI) commonly used for depression and anxiety therapy. Duloxetine is also frequently prescribed to cancer patients associated with depression symptoms [68]. It was recently reported that duloxetine could inhibit autophagic flux by downregulating AMPK phosphorylation in lung cancer cells [50] (Figure 2 and Table 2). The duloxetine-induced autophagy inhibition upregulated DR5 expression and enhanced TRAIL-mediated apoptosis, which indicated a promising approach for the TRAIL-resistant cancer therapy [50].

## 4. Anticancer Antidepressants Investigated for Combinational Treatment and in Clinical Trials

Antidepressants used alone have achieved much evidence in vitro and in vivo to demonstrated their anticancer properties, and they encompass tricyclic antidepressants (TCAs), tetracyclic antidepressants (TeCAs), selective serotonin reuptake inhibitors (SSRIs), and serotonin-norepinephrine reuptake inhibitors (SNRIs) [9,10,11]. Those anticancer antidepressants, which work through the mechanism of modulating autophagy, are summarized in Table 1 and Table 2.

A number of studies have also shown that the combination of antidepressants and conventional anticancer drugs increases effectiveness in cancer treatment. Desipramine fluoxetine, citalopram, and paroxetine could enhance the cytotoxicity of platinum drugs [69,70,71]. Fluoxetine, benztropine, fluphenazine, and paroxetine intensified the effects of paclitaxel or docetaxel to cancer [70,72,73,74]. A synergistic effect was found with the combination of 5-fluorouracil/doxorubicin and antidepressants including sertraline, thioridazine escitalopram, fluoxetine, imipramine, and paroxetine [70,73,74,75,76,77]. The combination of fluoxetine and raloxifene enhanced therapeutic effects in breast cancer [78,79]. Fluoxetine and imipramine synergized with temozolomide to induce significant cell death in glioblastoma [80,81]. Sertraline sensitized non–small cell lung cancer to erlotinib by inducing autophagy [40].

Although antidepressants have been demonstrated with sufficient preclinical evidence to have anticancer properties, further clinical trials are still needed to evaluate their clinical effect. We searched the ClinicalTrials.gov registry. As a result, we identified 11 registered clinical trials investigating the anticancer effects of autophagy-regulating antidepressants (Table 3). However, no interim or final results on their therapeutic effectiveness have been reported, which requires us to focus more efforts on clinical studies to confirm the anticancer potential of antidepressants.

## 5. Discussion

It is widely recognized that cancer is a global health problem. Current cancer treatment is often plagued by several major problems such as serious side effects, frequent therapy resistance, and the lack of effective drugs. Conventional chemotherapy and radiotherapy always cause serious toxicity to normal cells by triggering non-specific apoptosis, thus limiting their employment for cancer therapy. Treatment resistance also frequently occurs after a prolonged therapeutic cycle, along with new tumor colonies being developed and apoptosis-tolerant mutations being accumulated. Therefore, to develop new tumor-killing regimens independently relying on apoptotic cell death but additionally or alternatively working through other programed cell death mechanisms, like autophagy, should be a promising strategy to overcome potential treatment resistance. Furthermore, the lack of sufficient treatment options and effective drugs is a long-lasting obstacle facing clinical settings, especially for those less frequent cancer types [82]. Repurposing anticancer drugs from existing ones with the property of inducing autophagy-associated cell death, like antidepressants, represents a feasible way with cost-effective, time-saving, and less-toxicity advantages.

Autophagy plays crucial bidirectional roles in regulating cancer development. Either pharmacological induction or inhibition has been proven to effectively restrain tumor growth and metastasis. Therefore, to develop cancer therapeutics from autophagy modulators is considered to be a good strategy. Although there are several available small molecules that are specifically designed to modulate autophagy, none of these agents have completed clinical trials and been approved for clinical use so far. There is still a long process for the therapeutic application of novel autophagy modulators to treat cancer patients; it cannot begin until the completion of a full evaluation of efficacy, toxicity, pharmacokinetics, pharmacodynamics, and so on. Since developing novel drugs starting from the beginning is time-consuming and they are unavailable for clinical use soon, to repurpose existing clinical-used drugs with autophagy-modulating properties, such as the antidepressants reviewed in this study, represents a time-saving and cost-effective approach. Moreover, antidepressants have good advantages to penetrate the blood–brain barrier for being delivered to the brain, which is naturally suitable for treating intractable brain tumors such glioma and brain metastasis.

Herein, we reviewed autophagy-modulating antidepressants with antitumor effects. It was found that they targeted multiple autophagic processes, from the early stage associated with autophagosome formation to the late stage involved in lysosomal degradation (Figure 1 and Figure 2; Table 1 and Table 2). Among these antidepressant agents, sertraline, imipramine, desipramine; fluoxetine; vortioxetine, duloxetine, and clomipramine were reported to control the upstream signaling of autophagy via regulating AMPK or mTOR. Amitriptyline, nortriptyline, N-methylparoxetine, and paroxetine affect autophagy by managing the interplay between autophagosomes and lysosomes. Furthermore, maprotiline and fluoxetine also influence autophagy by modulating intracellular Ca^2+^ flux. During the past few decades, these drugs have been demonstrated with good safety profiles and frequently co-administrated with chemotherapeutic drugs for cancer patients. Recent studies have proven the anticancer properties of antidepressants through autophagy modulation. Thus, combining conventional chemotherapeutic regimens with antidepressants offers a promising anticancer treatment strategy to induce synergistic or additive tumor-killing effects by simultaneously triggering multiple programmed cancer cell death mechanisms.

There are still some unsolved tasks to be completed before the wide application of antidepressants for clinically treating cancer patients, although they have been well documented for suppressing tumor growth in animal experiments. Firstly, more intensive mechanism studies should be further conducted to elucidate how drug types and dosages modulate the anticancer effect, as well as to confirm their drug targets during the autophagy process. Several studies of anticancer antidepressants have merely observed an increase in the numbers of autophagic markers (Table 1 and Table 2) such as LC3II/I ratios; however, their results are not convincing for drawing certain conclusions about antidepressants’ functions with regard to autophagy. Antidepressants seem to affect autophagy in a number of ways, encompassing a variety of autophagy processes (Figure 1 and Figure 2). Varying concentrations of antidepressants administered in different experimental models may also affect their autophagic outcomes, which is another important consideration. Secondly, more extensive clinical trials are urgently required to evaluate the reliability and safety of repurposing antidepressants as anticancer drugs. There have been a number of studies demonstrating that antidepressants have anticancer properties. However, it has been suggested that certain antidepressants increase the risk of cancer development and recurrence [83]. A systematic review on the carcinogenicity of antidepressants found that 45% antidepressants (9/20 agents) were positive for carcinogenicity [84]. Many important anticancer targets are double-edged swords. It is necessary to find the balance between their advantages and disadvantages. In order to fully understand the clinical applicability of antidepressants to cancer patients, further research must be conducted. Thirdly, it is necessary to investigate the potential drug interaction between antidepressants and chemotherapeutic agents to provide a clear view showing whether the combination is potentially synergistic or antagonistic. Cytochrome P450 is responsible for most of the biological transformations of anticarcinogens [85]. If co-administered with antidepressants that inhibit this cytochrome P450 isoform, anticancer efficacy may be reduced or drug toxicity may be increased [85]. For example, tamoxifen is a kind of anticarcinogen for breast cancer that needs to be metabolized by cytochrome P450 2D6/CYP2D6. Meanwhile, some antidepressants (e.g., duloxetine and fluoxetine) are reported as strong inhibitors of CYP2D6 [86,87]. Therefore, some antidepressants should not be used in conjunction with tamoxifen.

Nevertheless, based on existing preclinical studies, we have reason to believe that antidepressants may potentially be developed as a promising therapeutic regimen to fight against cancer. To sum up, we introduced the anticancer potential of antidepressants and reviewed their underlying pharmacological mechanisms through the modulation of autophagic processes. We further discussed the prospects and limits of repurposing antidepressants as anticancer drugs.

## Figures and Tables

**Table 1:** Antidepressants with anticancer activity through inducing autophagy.

Antidepressant Types	Cancer Types	Cell Line	Models	Mechanism of Action	Ref.
Imipramine	Glioma	U-87MG	In vitro	Inhibits Akt/mTOR signaling	[26]
Imipramine	Glioma	LN-229; LN-71; LN-443	In vitro; in vivo	Increases cAMP levels	[27]
Imipramine	Glioblastoma	Primary glioblastoma cells	In vitro; in vivo	Induces autophagic flux	[28]
Desipramine	Glioma	C6	In vitro	Inhibits Akt/mTOR signaling; activates PERK-eIF2α-ER stress pathway	[29]
Maprotiline	Burkitt’s lymphoma	DG-75	In vitro	Increases Ca^2+^ influx	[30]
Amitriptyline	Hepatocellular carcinoma	HepG2	In vitro	Induces Parkin-dependent mitophagy	[31]
Fluoxetine	Burkitt’s lymphoma	DG-75	In vitro	Increases Ca^2+^ influx	[30]
Fluoxetine	Breast cancer	SUM149PT	In vitro	Activates AMPK and inhibits Akt/mTOR signaling; activates PERK-eIF2α-ER stress pathway	[32]
Fluoxetine	Breast cancer	MDA-MB-231; MDA-MB-436	In vitro	Activates AMPK and inhibits mTOR signaling; inhibits eEF2K signaling	[33]
Fluoxetine	Gastric cancer	AGS	In vitro	Inhibits Akt/mTOR signaling	[34]
Fluoxetine	Lung cancer	H460; A549	In vitro	Inhibits Akt/mTOR signaling	[35]
Escitalopram	Glioblastoma	GBM8401	In vitro; in vivo	Induces autophagy	[36]
Escitalopram	Hepatocellular carcinoma	HepG2; Huh-7	In vitro; In vivo	Induces autophagy	[37]
Sertraline	Acute myeloid leukemia	NB4	In vitro	Induces autophagy	[38]
Sertraline	Prostate cancer	Prostate cancer stem cells	In vitro	Induces autophagy	[39]
Sertraline	Lung cancer	A549; H522; PC9/R; H1975	In vitro; in vivo	Activates AMPK and inhibits mTOR/S6K signaling	[40]
Vortioxetine	Gastric cancer	AGS	In vitro	Inhibits AKT/mTOR signaling	[41]

**Table 2:** Antidepressants with anticancer activity through blocking autophagic flux.

Antidepressant Types	Cancer Types	Cell Line	Models	Mechanism of Action	Ref.
Desipramine	Melanoma	UACC903	In vitro; in vivo	Inhibits acid sphingomyelinase -mediated intracellular cholesterol transport	[42]
Amitriptyline	Lung cancer	A549	In vitro	Inhibits autophagosome–lysosomal fusion	[43]
Nortriptyline	Pineoblastoma	Primary pineoblastoma cells	In vitro; in vivo	Inhibits autophagosome–lysosomal fusion	[44]
Clomipramine	Prostate cancer	C4-2B	In vitro; in vivo	Inhibits autophagosome–lysosomal fusion	[45]
Norclomipramine	Cervical cancer	HeLa	In vitro	Blocks autophagic cargo degradation	[46]
Sertraline	Lung cancer	A549	In vitro	Inhibits AMPK phosphorylation	[47]
Paroxetine	Lung cancer	NCI-H1299; NCI-H1651	In vitro; in vivo	Inhibits lysosomal acidification	[48]
N-methylparoxetine	Lung cancer	NCI-H1299; NCI-H1650	In vitro	Inhibits lysosomal acidification and lysosomal cathepsins maturation	[49]
Duloxetine	Lung cancer	A549	In vitro	Inhibits AMPK phosphorylation	[50]

**Table 3:** Clinical trials using antidepressants in treating cancer patients.

Drugs	Study Description	Phase	Tumor Types	Start Date	ID
Imipramine	Imipramine on ER^+^ and triple-negative breast cancer	I	Breast cancer	July 2019	NCT03122444
Imipramine	Investigator-initiated study of imipramine hydrochloride and lomustine in recurrent glioblastoma	II	Glioblastoma	May 2022	NCT04863950
Desipramine	Phase 2a desipramine in small cell lung cancer and other high-grade neuroendocrine tumors	II	Small cell lung cancer; Neuroendocrine tumor	October 2012	NCT01719861
Maprotiline	A study of maprotiline in combination with tamoxifen and temozolomide for recurrent glioblastoma	I	Glioblastoma	June 2022	NCT04200066
Nortriptyline	Paclitaxel and nortriptyline hydrochloride in treating patients with relapsed small cell carcinoma	I	Small cell carcinoma	November 2016	NCT02881125
Fluoxetine	Combination chemotherapy plus fluoxetine in treating patients with advanced or recurrent non-small cell lung cancer	II	Lung cancer	August 2001	NCT00005850
Fluoxetine	Evaluation of fluoxetine and cytotoxic lysosomal stress in glioma (FLIRT)	I	Brain tumor	August 2023	NCT05634707
Escitalopram	Escitalopram to placebo in patients with localized pancreatic cancer	II	Pancreatic cancer	August 2022	NCT05289830
Sertraline	Sertraline and cytosine arabinoside in adults with relapsed and refractory AML	I	Acute myeloid leukemia	August 2016	NCT02891278
Sertraline	A proof-of-concept clinical trial assessing the safety of the coordinated undermining of survival paths by nine repurposed drugs combined with metronomic temozolomide for recurrent glioblastoma	II	Glioblastoma	November 2016	NCT02770378
Vortioxetine	Vortioxetine for MDD, cognition, and systemic inflammatory biomarkers	IV	Breast cancer	July 2016	NCT02637466

## Data Availability

No new data were created or analyzed in this study. Data sharing is not applicable to this article.

## Abbreviations

Akt: protein kinase B; AMPK, AMP-activated protein kinase; ATG, autophagy-related genes; Bak, BCL2 antagonist/killer; Bax, BCL2-associated X protein; Bcl-2, B-cell lymphoma-2; cAMP, Cyclic adenosine monophosphate; DR, Death receptor; eEF2K, eukaryotic elongation factor 2 kinase; eIF2α, Eukaryotic translation initiation factor 2A; ER, Endoplasmic reticulum; IARC, the International Agency for Research on Cancer; JNK, C-Jun kinase enzyme; LC3, microtubule-associated protein 1A/1B-light chain 3; MAPK, mitogen-activated protein kinase; mTOR, mammalian target of rapamycin; NF-κB, nuclear factor kappa-B; P2Y12, Purinergic receptor P2Y, G-protein coupled, 12; p62, Sequestosome 1; PERK, PRKR-like endoplasmic reticulum kinase; PI3K, phosphoinositide 3-kinase; S6K, p70 ribosomal S6 kinase; SNRI, Serotonin-norepinephrine reuptake inhibitor; SSRI, Selective serotonin reuptake inhibitor; TCA, Tricyclic antidepressant; TeCA, tetracyclic antidepressant; TRAIL, TNF-related apoptosis-inducing ligand; ULK, Unc-51-like autophagy-activating kinase; VEGF, vascular endothelial growth factor; Vps34, Vacuolar protein sorting 34.
